# Complete mitochondrial genome of *Eonemachilus longidorsalis* (Cypriniformes: Nemacheilidae): genome characterization and phylogenetic consideration

**DOI:** 10.1080/23802359.2023.2194459

**Published:** 2023-08-01

**Authors:** Lin Song, Xiao Jiang Chen, Qiu Wang, Quan Wang

**Affiliations:** Jiangsu Agri-Animal Husbandry Vocational College, Taizhou, P.R. China

**Keywords:** Mitochondrial genome, *Eonemachilus longidorsalis*, phylogenetic analysis

## Abstract

For the first time, this study presents the complete mitochondrial genome and phylogenetic analysis of *Eonemachilus longidorsalis*, an endemic species in southwest China. The mitogenome is a circular molecule of 16,569 bp in length with a base composition of 30.2% A, 27.3% T, 16.6% G, and 25.9% C. The phylogenetic analysis based on the complete mitogenomes shows that *E. Longidorsalis* clusters with *Yunnanilus jiuchiensis*, *Yunnanilus pleurotaenia* and *Eonemachilus niger.* This contribution may provide a valuable framework for completely resolving phylogenetic relationships of the family Nemacheilidae in future research.

## Introduction

*Eonemachilus longidorsalis* (Li, Tao and Lu 2000) is distributed in the eastern Yunnan, Nanpangjiang drainage of China. This species combines morphological characteristics that can be distinguished from other *Eonemachilus* fishes: scales covering the whole body; extremely long dorsal fin iii-11-12; anal fin ii-7; pectoral fin i-10; caudal fin i-14-i; 12 inner gill rakers on the first gill-arch and no outer gill rakes (Du et al. 2021). *Eonemachilus* (Berg 1938) was once regarded as a synonym of *Yunnanilus* by some ichthyologists (Kottelat and Chu 1988; Zhu 1989; Yang 1991), but morphological studies supported that *Eonemachilus* was a valid genus, which can be distinguished from *Yunnanilus* based on absent (vs. present) lateral line and cephalic lateral-line canals (Li WX et al. 2000; Kottelat 2012). This viewpoint was confirmed by the evolutionary position of *E. nigomaculatus* based on molecular data, which was the most basal clade of the tribes Lefuini, Nemacheilini, Triplophysini, and some members of Yunnanilini (Du et al. 2021). However, we noticed that the sequence of *E. nigomaculatus* was misidentified and changed in GenBank by the author, resulting in the invalidity of previous phylogenetic analyses conducted from molecular perspective. In this study, the complete mitochondrial genome of *E. longidorsalis* was determined and described, and its taxonomic status in Nemacheilidae fishes was analyzed, which may provide a valuable genetic resource for further phylogeny analysis on the genus *Eonemachilus.*

## Materials

**Figure 1.**
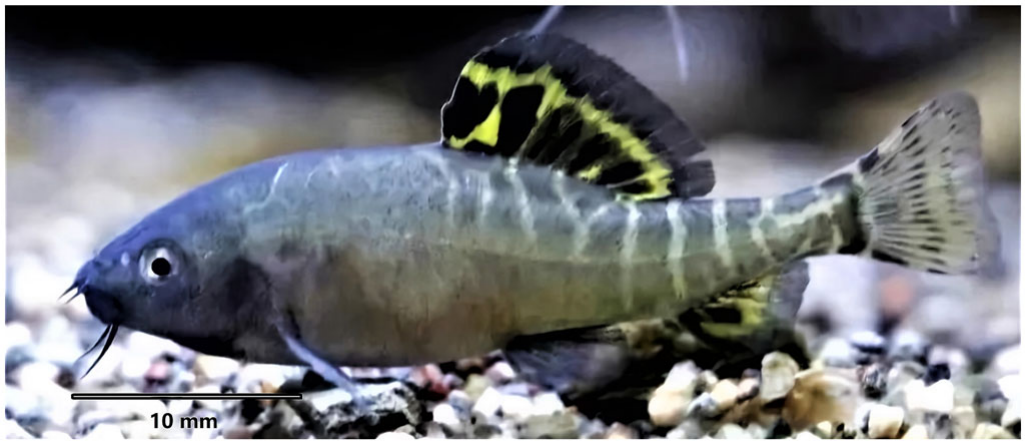
The specimen of *Eonemachilus longidorsalis* from Nanpangjiang, Qujing, Yunnan Province, China (Photo by Lin Song).

The *E. longidorsalis* specimen was collected from Nanpangjiang, Qujing, Yunnan Province of China (25°58′76.38″N, 103°83′05.79″E), and it was identified according to morphological keys (Figure 1) (Du et al. 2021). Then the sample was preserved in 95% ethanol and deposited in Aquatic Science and Technology Institution Herbarium under the voucher number ASTIH-21b1108d27 (https://www.jsahvc.edu.cn/; person in charge of the collection: Lin Song; email: tianxinlinger@126.com).

## Methods

After being transported to Shanghai Genesky Biotechnologies Inc, total genomic DNA was extracted from muscle using the Tguide Cell/tissue genomic DNA Extraction Kit (OSR-M401) (Tiangen, Beijing, China). The next several steps were DNA sample quality control, DNA library construction, PCR amplification, size selection, library quality check, and library pooling. Sequencing was conducted on the Illumina HiSeq 4000 Sequencing platform (Illumina, CA, USA). The complete mitogenome of *E. longidorsalis* was obtained by sequence assembly on MetaSPAdes software (Nurk et al. 2017). The annotation process was executed using MitoMaker (Bernt et al. 2013). The mitogenome sequence and gene annotations were submitted to the National Center for Biotechnology Information GenBank database under the accession number OM732331. The CGView online server (https://proksee.ca/) was chosen to draw the mitogenome map.

To infer the taxonomic status of *E. longidorsalis*, the complete mitochondrial genomes of 31 Nemacheilidae species and 2 Balitoridae species were concatenated into a dataset, and a phylogenetic tree was constructed *via* the maximum likelihood method under the most suitable nucleotide sequence model GTR + G+I on MEGA X (Kumar et al. 2018). The 2 Balitoridae species, *Sinogastromyzon szechuanensis* (Li W et al. 2021) and *Metahomaloptera omeiensis* (Li Y et al. 2016) were used as outgroups.

## Results

### Mitogenome organization

**Figure 2.**
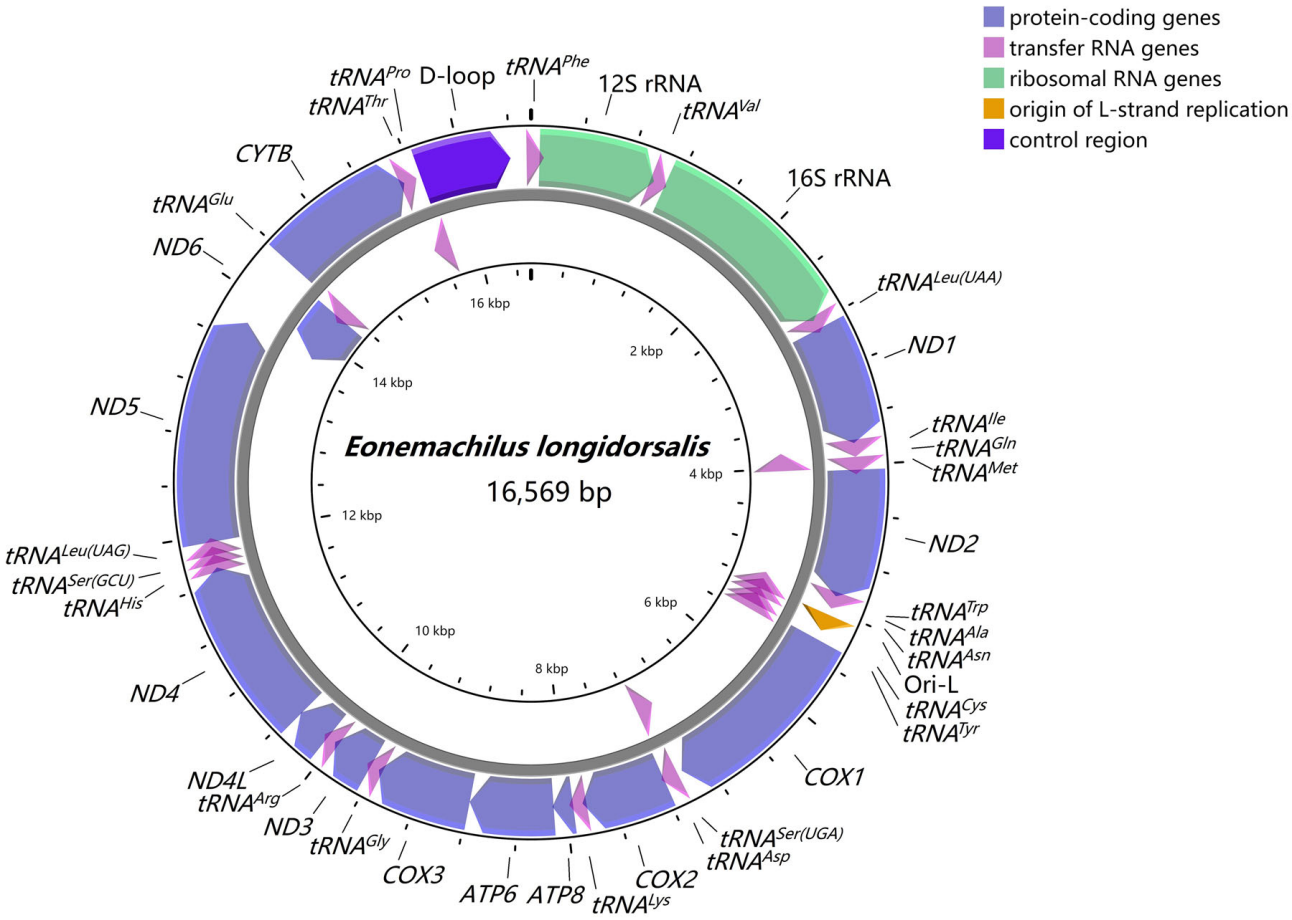
Gene map of the mitochondrial genome of *Eonemachilus longidorsalis*. Gene encoded on H- and L- strands with inverse arrow directions were shown outside and inside the circle, respectively. The complete mitogenome of *E. longidorsalis* is 16,569 bp with the inclusion of 13 protein-coding genes, 22 transfer RNA genes, two ribosomal RNA genes, origin of L-strand replication (Ori-L) and control region (D-loop).

The circular mitogenome is 16,569 bp in length, encompassing 13 protein-coding genes (PCGs), two ribosomal RNA (rRNA) genes, 22 transfer RNA (tRNA) genes, and a control region (D-loop) (Figure 2). The nucleotide composition of *E. longidorsalis* appears to be 30.2% A, 27.3% T, 16.6% G, and 25.9% C, with an AT bias of 57.5%. Most genes are encoded on the H-strand except for *ND6* and 8 tRNA genes (*tRNA^Gln^*, *tRNA^Ala^*, *tRNA^Asn^*, *tRNA^Cys^*, *tRNA^Tyr^*, *tRNA^Ser(UCN)^*, *tRNA^Glu^*, and *tRNA^Pro^*). 22 transfer RNA genes range from 67 to 76 bp in length. The large ribosomal gene (16S) located between *tRNA^Val^* and *tRNA^Leu(UUR)^* is 1,657 bp long, and the small (12S) located between *tRNA^Phe^* and *tRNA^Val^* is 953 bp long. Furthermore, the noncoding control region located between *tRNA^Phe^* and *tRNA^Pro^* is 745 bp in length. Among the 13 protein-coding genes, the lengths vary from 168 bp (*ATP8*) to 1,839 bp (*ND5*). All PCGs conventionally utilize ATG as the start codon with the exception of *COX1*, which starts with GTG. 3 PCGs (*COX2*, *ND4*, and *CYTB*) end with a single nucleotide T, and *COX3* sets incomplete TA as the stop codon. 6 PCGs (*ND1*, *COX1*, *ATP8*, *ATP6*, *ND4L*, and *ND5*) terminate with canonical TAA, while 3 PCGs (*ND2*, *ND3*, and *ND6*) end with complete TAG. Sequence analyses display that the mitogenome has 2 overlaps between tRNAs (*tRNA^Ile^*-*tRNA^Gln^*, *tRNA^Thr^*-*tRNA^Pro^*), and 4 overlaps between protein-coding genes (*ATP8-ATP6*, *ATP6-COX3*, *ND4L-ND4*, *ND5-ND6*).

### Phylogenetic analysis

**Figure 3.**
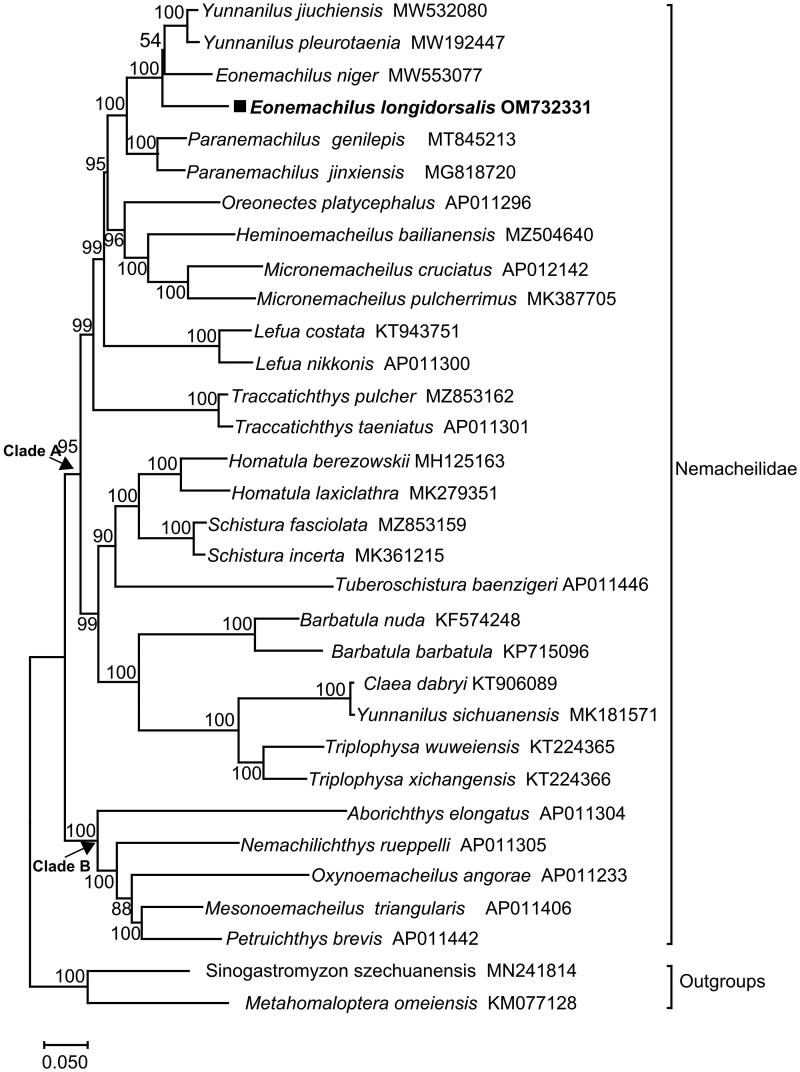
Maximum-likelihood (ML) phylogenetic tree reconstructed using concatenated mitochondrial genomes of *E. longidorsalis* and other 32 fishes. Accession numbers are indicated after the species names. The tree topology was evaluated by 1000 bootstrap replicates. Bootstrap values at the nodes correspond to the support values for ML methods.

As shown in Figure 3, based on the complete mt DNA genome sequences of 33 species, the family Nemacheilidae is divided into two clades: 14 genera (*Yunnanilus*, *Eonemachilus*, *Paranemachilus*, *Oreonectes*, *Heminoemacheilus*, *Micronemacheilus*, *Lefua, Traccatichthys*, *Homatula*, *Schistura*, *Tuberoschistura*, *Barbatula*, *Claea* and *Triplophysa*) form Clade A, while 5 genera (*Aborichthys*, *Nemachilichthys*, *Oxynoemacheilus*, *Mesonoemacheilus* and *Petruichthys*) form Clade B. Meanwhile, *E. longidorsalis* is clustered with *Yunnanilus jiuchiensis* (Du et al. 2021), *Yunnanilus pleurotaenia* and *Eonemachilus niger.* Notably, *Yunnanilus sichuanensis* is clustered with *Claea dabryi*, which indicates that the genus *Yunnanilus* is complexible.

## Discussion and conclusion

Through next-generation sequencing and assembly, the complete mitogenome of *E. longidorsalis* is 16,569 bp in length. The gene order and composition are identical to typical mitogenomes of other teleost fishes (Luo et al. 2019). The maximum likelihood tree based on the complete mitochondrial genomes of *E. longidorsalis* and 32 other species supports that *E. longidorsalis* constitutes a sister-group mitogenome relationship with *Yunnanilus jiuchiensis*, *Yunnanilus pleurotaenia* and *Eonemachilus niger.* The inclusion of more related taxa on future whole mitogenome phylogenetic analyses may help to understand the phylogeny of *Yunnanilus* and *Eonemachilus* (Yamasaki et al. 2015; Wang et al. 2019; Maeda et al. 2021). In conclusion, this study will provide important information for future taxonomic, systematic, and genetic studies of Nemacheilidae.

## Data Availability

The genome sequence data that support the findings of this study are openly available in GenBank of NCBI at (https://www.ncbi.nlm.nih.gov/) under the reference number OM732331. The associated ‘BioProject,’ ‘Bio-Sample’ and ‘SRA’ numbers are PRJNA816066, SAMN26656524, and SRR18326284 respectively.
